# Immunometabolism: Molecular Mechanisms, Diseases, and Therapies

**DOI:** 10.1155/2014/585708

**Published:** 2014-09-22

**Authors:** José Cesar Rosa Neto, Fábio Santos Lira, William Tadeu Festuccia

**Affiliations:** ^1^Immunometabolism Research Group, Institute of Biomedical Sciences, University of São Paulo (USP), 05508-000 São Paulo, SP, Brazil; ^2^Exercise and Immunometabolism Research Group, Department of Physical Education, Universidade Estadual Paulista (UNESP), 19060-900 Presidente Prudente, SP, Brazil; ^3^Department of Physiology and Biophysics, Institute of Biomedical Sciences, University of São Paulo (USP), 05508-000 São Paulo, SP, Brazil

Several studies published over the last two decades have provided a good body of evidences supporting a central role of chronic low-grade inflammation as a major factor driving many of the metabolic complications commonly found in highly prevalent chronic diseases such as obesity, insulin resistance, and cancer. It was well established in these studies, for example, that activation of canonical inflammatory pathways is one of the major factors promoting the impairment in insulin signaling seen in obesity and type 2 diabetes, being responsible for the reduced glucose uptake and exacerbated lipolysis found in this condition. Such important role of inflammation in chronic diseases has motivated several studies aiming at understanding the mechanisms underlying its development and searching for efficient therapeutic strategies to minimize its metabolic consequences. In the present special issue, we gathered several original and review articles addressing potential nutritional, pharmacological, and behavioral strategies that could be used to counteract inflammatory process associated with a variety of diseases including obesity, periodontal disease, myocardial infarction, and pneumonia, among others. In a very interesting study, for example, E. G. Novoselova et al. experimentally tested individually or in combination several inhibitors of NFkB pathway and naturally occurring antioxidants as anti-inflammatory agents in vivo bringing new information about their therapeutic efficacy. In the same direction, M. S. Elburki et al. tested the appropriateness of using a novel chemically modified curcumin, a matrix metalloproteinase inhibitor with no antibiotic properties, as a therapeutic molecule for the treatment of periodontal disease with promising results towards the attenuation of alveolar bone loss.

In addition to the above-mentioned pharmacological agents, there are in this special issue two interesting studies reporting promising effects of nutrients and vitamins as anti-inflammatory molecules in vivo. In a very elegant study, Y. Chen et al. report the beneficial actions of vitamin C supplementation in the attenuation of the inflammation and oxidative stress induced by LPS in macrophages from humans with community-acquired pneumonia. Also important were the findings of C. O. Souza et al. showing that a supplementation with the monounsaturated palmitoleic acid markedly attenuates obesity-associated fat accumulation, inflammation, and insulin resistance in the liver, such effects that do not depend on the nuclear receptor peroxisome-proliferator activated receptor (PPAR) alpha.

As detailed in the three different manuscripts discussed below, behavioral strategies such as exercise training were also addressed in this special issue, as strategies to attenuate inflammatory processes. A. Correia et al., for example, report in an original study interesting effects of exercise training in different intensities as an attenuator of the changes induced by aging in skeletal muscle inflammation and metabolism. This study is followed by two comprehensive review articles addressing the effects of exercise training on inflammation and autonomic dysfunction induced by myocardial infarction (B. Rodrigues et al.) and on lymphocyte metabolism and function in several conditions (F. Wasinski et al.). Finally, completing this special issue are the exciting findings of C. R. Balistreri et al. that report a strong association between toll-like receptor rs4986790 TLR4 polymorphism with development of type 2 diabetes in humans adding further support to the previous recognized role of this inflammatory pathway in the development of insulin resistance and S. Y. Gun et al. that extensively reviewed the role of interferons and regulatory factors as major components of the innate and adaptive responses fighting against malaria infection.

Overall, we believe that this special issue provides new insights into the complex interactions between inflammatory processes and underlying metabolic disarrangements commonly found in chronic diseases such as obesity, type 2 diabetes, and cancer, with an especial emphasis on the different pharmacological, nutritional, and behavioral strategies that could be used to prevent and/or attenuate such deleterious interrelationship.

*José Cesar Rosa Neto* *José Cesar Rosa Neto* *Fábio Santos Lira* *Fábio Santos Lira* *William Tadeu Festuccia* *William Tadeu Festuccia*

